# Sequential Utilization of E-space for Correction of Moderate Crowding: A Case Report

**DOI:** 10.5005/jp-journals-10005-1568

**Published:** 2018

**Authors:** Munish Reddy, Shalu Jain, Pradeep Raghav, Stuti Mohan, Ashutosh Wadhawan

**Affiliations:** 1-5 Department of Orthodontics and Dentofacial Orthopedics, Subharti Dental College, Swami Vivekanand Subharti University, Meerut, Uttar Pradesh, India

**Keywords:** E-space utilization, Late mixed dentition, Orthodontics, Tooth movement

## Abstract

Leeway space and E-space plays a crucial role in the late transition phase from primary dentition to permanent dentition. Leeway space is normally 0.9 mm and 1.5 mm in upper and lower arch respectively. E-space is the difference in the mesiodistal width of deciduous second molar and permanent second premolar which is 2.3 in upper arch 2.5 mm in lower arch 1. E-space and leeway space are normally utilized by permanent first molars to establish class I molar relation in permanent dentition. We can also utilize this E-space during fixed orthodontic treatment for reliving of crowding in late mixed dentition. Thus, here in this article, we are presenting clinical technique along with case series of sequential utilization of E-space for correction of anterior crowding without extractions.

**How to cite this article:**

Reddy M, Jain S, Raghav P, Mohan S, Wadhawan A. Sequential Utilization of E-space for Correction of Moderate Crowding: A Case Report. Int J Clin Pediatr Dent, 2018;11(6):519-525

## CASE REPORT 1

**Figs 1A and B:**
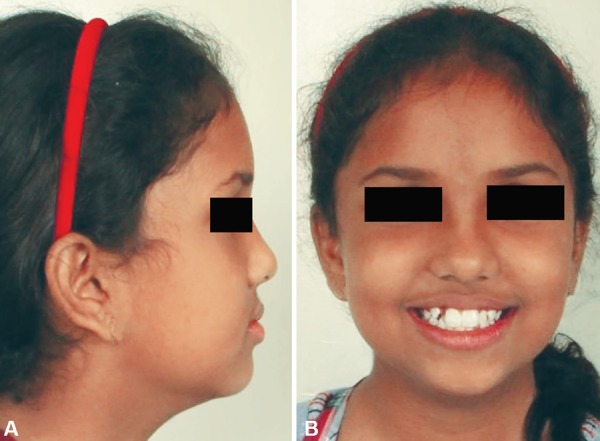
Extraoral photographs showing straight profile and irregular front teeth

**Figs 2A and B:**
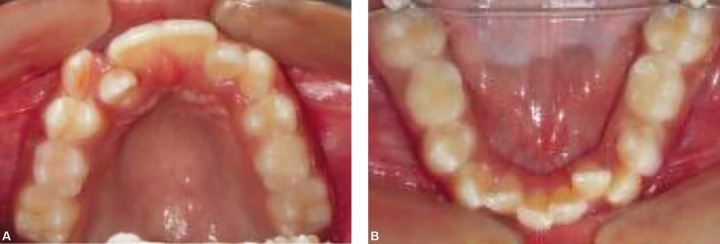
Intraoral photographs showing late mixed dentition and crowding in both the arches

**Fig. 3:**
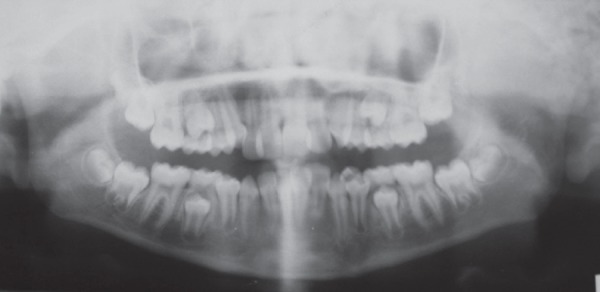
Orthopantomogram showing permanent second premolars in various stages of the eruption

**Figs 4 and B:**
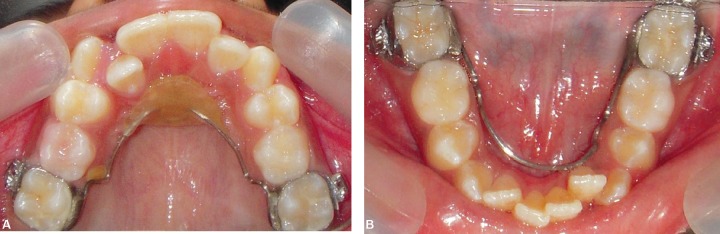
Occlusal photographs showing nance palatal arch and lingual arch

**Figs 5A and B:**
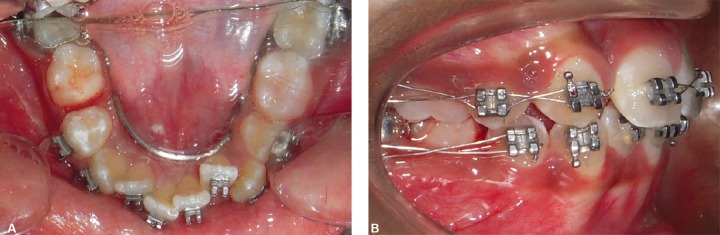
Intraoral photographs showing sequential reduction of E from mesial surface

**Figs 6A and B:**
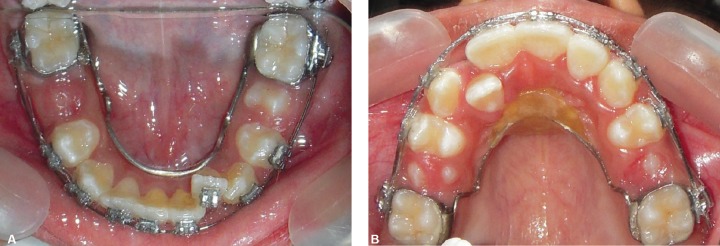
Occlusal photographs showing arch alignment at the time of E shedding

**Figs 7A to D:**
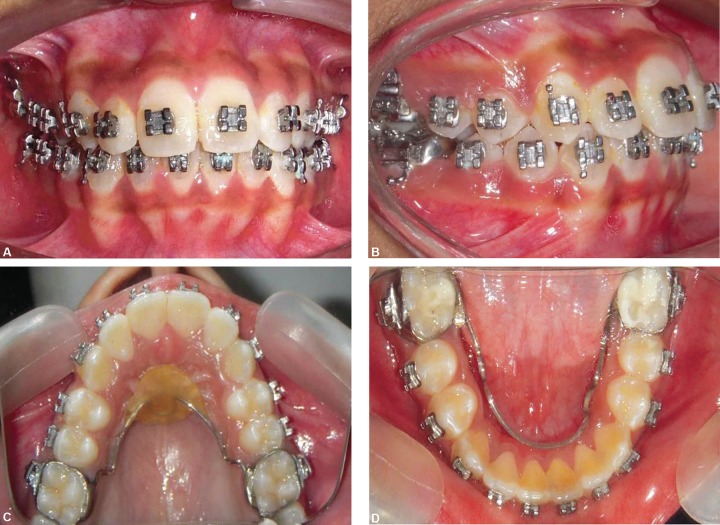
Intraoral photographs after treatment

**Figs 8A and B:**
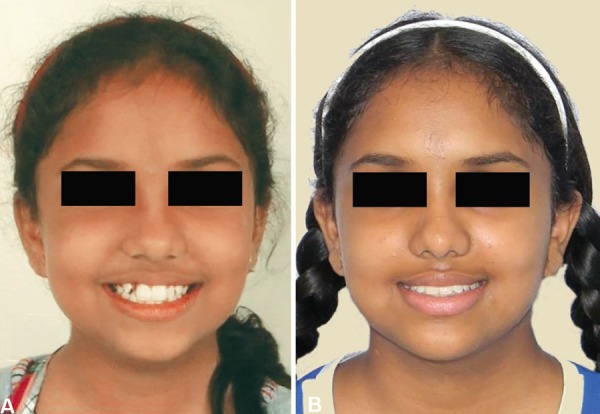
Extraoral frontal photographs after treatment

**Figs 9A and B:**
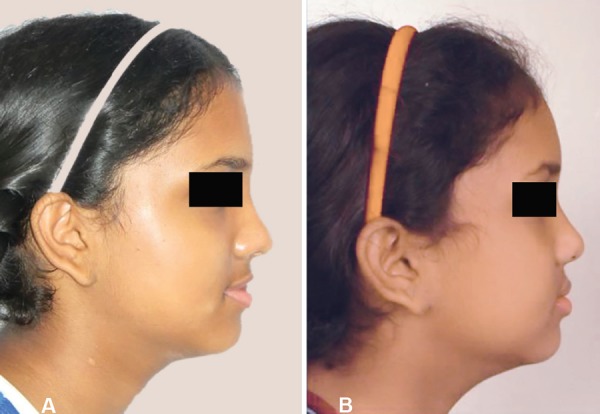
Extraoral photographs after treatment showing a pleasant smile and esthetic profile

A 9-year-old female patient born to nonconsanguineous parents reported to the Department of Orthodontics and Dentofacial Orthopaedics, Subharti Dental College, Meerut, with a chief complaint of irregularly placed upper and lower front teeth. On extraoral examination (Fig. 1), she was having a straight profile, unesthetic smile with a prominent maxillary canine on the right side. Intraoral examination (Fig. 2) revealed that she was in the late mixed dentition stage with Angle's class I molar relation on both sides. Maxillary midline was shifted to the right by 1.5 mm with respect to facial midline, and mandibular midline was shifted to left by 1 mm. All deciduous second molars (E's) were present. Maxillary right lateral incisor was in crossbite. Her radiological examination (Fig. 3) revealed that all permanent second premolars with 2/3rd of their roots completed were developing in between roots of deciduous second molars. Orthodontic records were prepared. Her model analysis revealed 6.5 mm and 7 mm tooth material excess in upper and lower arches, respectively. Based on her dental age and model and cephalometric analysis, it was decided to use E-space for correction of crowding in anterior teeth and guided eruption of permanent second premolars. Nance's palatal arch and lingual holding arch were constructed and cemented in upper and lower arches, respectively (Fig. 4). Arch alignment was started with partial bonding in both arches. E's were sequentially reduced at the rate of 1 mm/month from the mesial side for distalization of first premolars and canines with tie backs in all four quadrants (Fig. 5). Alignment was improved significantly in both arches till the time of shedding of Es (Fig. 6). We were able to align both the arches completely without any significant proclination of incisors. Molars were maintained in class I relationship, and canines were brought into class I relationship (Fig. 7). There was perfect alignment as seen in occlusal photographs (Fig. 8). Profile of the patient was satisfactory after treatment (Fig. 9).

## CASE REPORT 2

**Figs 10A and B:**
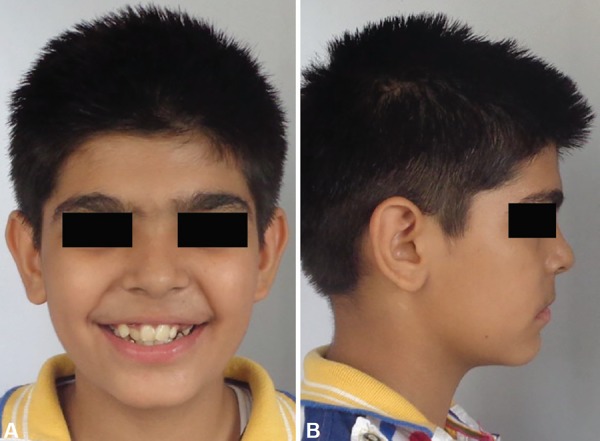
Extraoral photographs showing irregular front teeth and straight profile

**Figs 11A and B:**
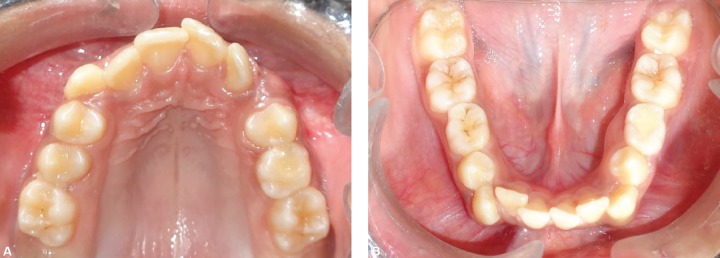
Intraoral photographs showing late mixed dentition and crowding in both the arches

**Fig 12:**
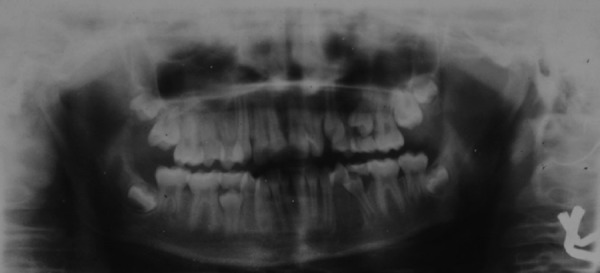
Preteatment orthopentogram

**Figs 13A and B:**
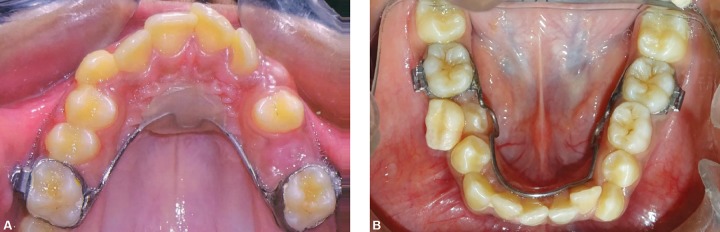
Occlusal photographs showing Nance's palatal arch and lingual arch

**Figs 14A and B:**
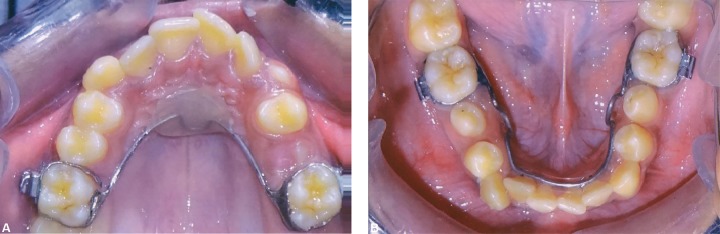
Occlusal photographs after 4 months of anchorage preparation

**Figs 15A to D:**
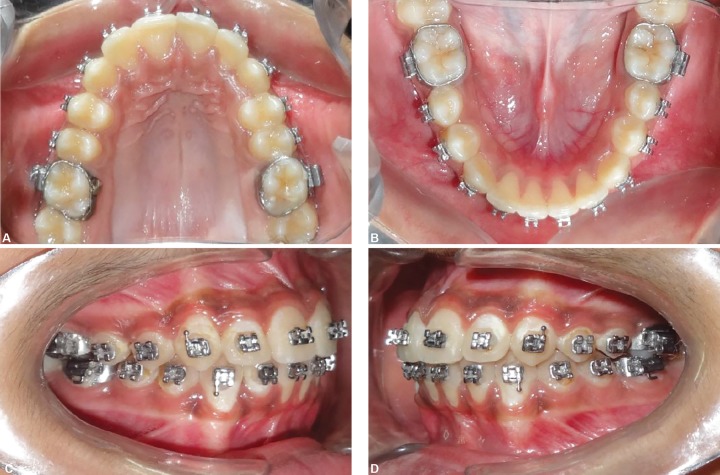
Intraoral photographs after treatment

**Figs 16A and B:**
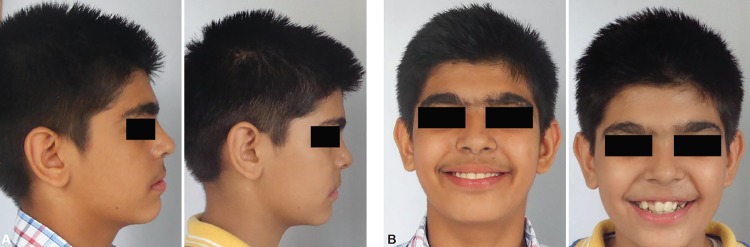
Comparison of extraoral profile and frontal photographs after treatment

An 11-year-old male patient reported to the Department of Orthodontics and Dentofacial Orthopedics, Subharti Dental College, Meerut, with a chief complaint of irregularly placed upper and lower front teeth. On examination extra orally (Fig. 10), the patient had a straight profile and competent lips. Intraoral examination (Fig. 11) revealed that he was in the late mixed dentition stage with Angle's class I molar relation on both sides. His mandibular midline was shifted to the right by 2.5 mm. Both maxillary right and left lateral incisors were in crossbite. The permanent upper left canine was missing. Deciduous upper left and lower right and left molars were present. His radiological examination (Fig. 12) revealed that upper left and lower both right and left permanent second premolars with 2/3rd of roots completed were developing in between roots of deciduous second molars. Orthodontic records were prepared. His model analysis revealed 4 mm and 2 mm tooth material excess in upper and lower arches respectively. As done previously, based on his dental age and model and cephalometric analysis, it was decided to use E-space for correction of crowding in anterior teeth and guided eruption of permanent second premolars. Nance's palatal arch and lingual holding arch were made and cemented in upper and lower arches respectively (Fig. 13). Partial bonding was done, and arch alignment was initiated. E's space was maintained using Nance's palatal arch and lingual holding arch as during treatment the deciduous molars got exfoliated. Canines were tied back in all four quadrants. Alignment was improved significantly in both arches. We were able to align both the arches completely without any significant proclination of incisors. Molars were maintained in class I relationship, and canines were brought into class I relationship (Fig. 14). There was perfect alignment as seen in occlusal photographs (Fig. 15). Profile of the patient was satisfactory after treatment (Fig. 16).

## DISCUSSION

Retained deciduous second molar affects much of the orthodontic treatment. The orthodontist must make the proper decision at the appropriate time regarding management of the E-space. E-space can be utilized either for correction of anterior crowding during leveling and alignment phase or would be eventually lost by mesial migration of first permanent molar. Most of the case reports^1–4^ in literature have discussed the management of congenitally missing permanent second premolars. As per best of our knowledge, no case report/article has discussed the utilization of E-space for relieving of anterior crowding along with fixed mechanotherapy.

So, here we have presented two options for utilization of the E-space with distalization of first premolar and canine simultaneously. Firstly, it can be done either by reduction of second primary molar sequentially or secondly, by maintaining the E-space till the eruption of the second premolar with the help of space maintainers. In the first case, the primary second molar was sequentially reduced. Usually, mandibular primary second molars are approximately 10–12 mm wide. By reducing the mesiodistal width, the tooth can be narrowed to about 8 mm. Also by retaining the primary second molar, but reducing its width and simultaneously distalizing the first premolar and canine into that space, we were able to gain space which was further used for relieving of anterior crowding with fixed mechanotherapy. The reduction of a deciduous molar should be accomplished with a sharply tapered carbides sure bur. The key is to remove sufficient tooth structure to create space but not enough to cause pulpal necrosis. A guide to estimate the correct amount of reduction is to measure the mesiodistal width of the deciduous molar at the level of the cementoenamel junction on a bitewing radiograph. This distance was transferred to and marked on the occlusal surface of the deciduous molar with a pencil or marking pen. Then the bur was positioned to follow this line and cut toward the gingiva to remove a wafer of enamel and the underlying dentin on both the mesial and distal surfaces. About 2 mm was removed from both surfaces; this left the crown about 7–8 mm wide.^5^ As space was created, canine and premolars were tied with active lacebacks and now fixed treatment was started during late mixed dentition itself and this space was utilized for correction of mild to moderate crowding. This helped in the conversion of a potential extraction case into nonextraction case as seen in our case report.

In the second case, the deciduous second molar was about to get exfoliated on the right side along with 1/4th erupted the first premolar whereas it was retained on the left side. It was decided to preserve the E-space in the lower arch with the help of lingual arch and on left side retained deciduous molar acted as a natural space maintainer. As in the previous case, lacebacks were given on canine, and leveling and alignment was continued. Soon the deciduous molar got exfoliated from both sides, and the anterior teeth were aligned with relieving of crowding just by preserving and utilizing the E-space. This case was also converted from extraction to nonextraction case.

## CONCLUSION

In the late mixed dentition stage, we should keep a check on the erupting permanent and retained primary teeth because with sequential utilization of E-space, we can convert potential extraction case into nonextraction category. This gives an advantage of shortening total duration time and reducing patient anxiety related to extraction.
